# Comprehensive analysis to construct a novel immune-related prognostic panel in aging-related gastric cancer based on the lncRNA‒miRNA-mRNA ceRNA network

**DOI:** 10.3389/fmolb.2023.1163977

**Published:** 2023-05-15

**Authors:** Cuncan Deng, Juzheng Peng, Cheng Yuan, Huafu Li, Wenchao Li, Hongwu Chu, Hongfa Wei, Yulong He, Leli Zeng, Mingyu Huo, Changhua Zhang

**Affiliations:** ^1^ Digestive Diseases Center, The Seventh Affiliated Hospital, Sun Yat-sen University, Shenzhen, China; ^2^ Department of Thoracic Surgery, Zhujiang Hospital, Southern Medical University, Guangzhou, China; ^3^ Institute of Cancer Research, Cancer Stem Cell Team, London, United Kingdom; ^4^ Guangdong Provincial Key Laboratory of Digestive Cancer Research, The Seventh Affiliated Hospital of Sun Yat-sen University, Shenzhen, Guangdong, China; ^5^ The Seventh Affiliated Hospital, Sun Yat-Sen University, Shenzhen, China

**Keywords:** gastric cancer, ceRNA network, prognostic panel, immune infiltration, lncRNA

## Abstract

**Introduction:** Gastric cancer (GC) is the fifth frequent malignancy and is responsible for the third leading cause of cancer-related deaths. Gastric cancer is an aging-related disease, with incidence and mortality rates increasing with aging. The development of GC is affected by lncRNAs, miRNAs, and mRNAs at the transcriptional and posttranscriptional levels. This study aimed to establish a prognostic panel for GC based on competing endogenous RNA (ceRNA) networks.

**Methods:** RNA sequences were obtained from the TCGA database. Different expressions of RNAs were scrutinized with the EdgeR package. The ceRNA network was built using the starBase database and the Cytoscape. The prognostic panel was constituted with the LASSO algorithm. We developed a nomogram comprising clinical characteristic and risk score. The receiver operating characteristic (ROC) was used to evaluate the accuracy of the nomogram prediction. Hub RNAs expressions were detected by qPCR, immunohistochemistry and western blot respectively. Clinical relevance and survival analyses were analyzed. The relationship between RNAs and immune infiltrations, as well as immune checkpoints, was analyzed and evaluated using the CIBERSORT, TIMER and TISIDB databases.

**Results:** Four DElncRNAs, 21 DEmiRNAs and 45 DEmRNAs were included in the ceRNA network. A 3-element panel (comprising lncRNA PVT1, hsa-miR-130a-3p and RECK) with poor overall survival (OS) was established and qPCR was applied to validate the expressions of hub RNAs. Hub RNAs were firmly associated with T, M, and N stage. The CIBERSORT database showed that the high lassoScore group exhibited a significantly high ratio of resting memory CD4^+^ T cells, M2 macrophages and a significantly low ratio of activated memory CD4^+^ T cells and M1 macrophages. According to the TIMER database, this panel was linked to immune infiltrations and immune cell gene markers. TISIDB database indicated that RECK was positively correlated with immune checkpoints (including CD160, CD244, PDCD1, and TGFBR1).

**Discussion:** A novel triple prognostic panel of GC constructed based on the ceRNA network was associated with clinical prognostic, clinicopathological features, immune infiltrations, immune checkpoints and immune gene markers. This panel might provide potential therapeutic targets for GC and more experimental verification research is needed.

## 1 Introduction

Aging is defined as the gradual loss of physiological integrity, accompanied by diminished function and elevated vulnerability to death. (López-Otín et al., 2023a). It is one of the important risk factors for many types of cancer, with the incidence increasing with age and reaching a peak at age 85 (White et al., 2014). Gastric cancer (GC) is the fifth frequent malignancy around the world causing the third cancer-related death worldwide (Smyth et al., 2020). The incidence of gastric cancer increases with age. In China, the incidence of GC in all age groups was less than 1/100,000 before the age of 25, and it was more than 50/100,000 from the age of 55 and reached its peak at the age of 80–84, which was 185.85 per 100,000(4). More than 1 million people have been diagnosed with GC and 784000 deaths were caused by GC worldwide in 2018 (Bray et al., 2018). Although some progress has been achieved in both the diagnosis and therapy of GC, the overall survival (OS) ratio is still unsatisfactory, below 30% in many countries (Allemani et al., 2015). The current dilemma of gastric cancer includes the lack of effective early diagnosis, poor clinical outcomes, and high metastasis and recurrence rates. Elucidating the pathogenesis and identifying effective biomarkers of gastric cancer are meaningful for improving the diagnosis, prevention and therapy of GC.

Several characteristics of aging and cancer are very similar and are defined as meta-hallmarks, including epigenetic alterations, chronic inflammation, genomic instability, and dysbiosis (López-Otín et al., 2023b). The characteristics of aging may be the promoters of tumors. mRNAs and ncRNAs, including lncRNAs and microRNAs (miRNAs), affect aging and cancer by targeting multiple components of longevity and oncogenic pathways at the transcriptional and post-transcriptional levels (Slack and Chinnaiyan, 2019; López-Otín et al., 2023b). Long noncoding RNAs (lncRNAs) are the transcripts containing more than two hundred nucleotides and could not be translated into proteins (Kopp and Mendell, 2018). Aberrant expressions of lncRNAs are firmly related to tumorigenesis, metastasis, and tumor stage (Vitiello et al., 2015; Bhan et al., 2017). Studies have proved that lncRNAs could act as effective diagnostic and prognostic biomarkers in GC (Fattahi et al., 2020). According to the competitive endogenous RNA (ceRNA) hypothesis, lncRNAs mainly influence the expression of mRNAs through binding to miRNAs in cancers (Salmena et al., 2011).

MicroRNAs (miRNAs) are another type of short single-stranded non-coding RNA (ncRNAs), possessing a length of 19–25 nucleotides (Lu and Rothenberg, 2018). MiRNAs can silence certain genes on the posttranscriptional level via binding to the 3′-untranslated region (3′-UTR) in a miRNA response element (MRE) sequence (Lu and Rothenberg, 2018; Ouyang et al., 2021). miRNAs are essential in the whole stage of GC, comprising diagnosis, oncogenesis, development, treatment and prognosis (Ouyang et al., 2021). Dysregulated miRNAs can promote or inhibit tumors by targeting different mRNAs in diverse signal pathways involved in migration, invasion, cell proliferation and angiogenesis (Yang et al., 2014; Tsai et al., 2016; Alessandrini et al., 2018).

Competitive endogenous RNAs can influence each other at the post-transcriptional level via competingly binding to commensal miRNAs. MicroRNAs can cause gene silencing via binding mRNAs, while ceRNAs (such as lncRNAs) can adjust mRNAs expression by competitively combining microRNAs through MREs (Salmena et al., 2011). LINC01133 inhibited GC progression via binding to miR-106a-3p to adjust the expression of APC and to influence the Wnt signal pathway (Yang et al., 2018b). LncRNA MT1JP regulates the function of FBXW7 in GC by competitively binding miR-92a-3p (Zhang et al., 2018). MEF2A-mediated lncRNA HCP5 suppresses GC proliferation through the MiR-106b-5p/p21 axis (Chen et al., 2021).

The aberrant expression of lncRNAs, mRNAs, and miRNAs influences the occurrence and progression of GC at different RNA levels (Zhang et al., 2020). Mapping the lncRNA‒miRNA-mRNA ceRNA network in GC is critical for understanding its pathogenesis. The construction of a prognostic panel utilizing a competing endogenous RNA (ceRNA) network has the potential to provide a more thorough and precise evaluation of the clinical prognostic significance of GC.

In this study, the objective was to describe the landscape of the ceRNA network and build a novel prognostic panel in GC. First, miRNA-seq data and RNA-seq of GC were obtained from The Cancer Genome Atlas (TCGA) database. RNA interactions of lncRNAs, miRNAs and mRNAs were predicted by the StarBase database and a GC-related ceRNA network was built using Cytoscape. Then, the least absolute shrinkage and selection operator (LASSO) algorithm was utilized to build a prognostic panel with hub RNAs involved in the ceRNA network. Cox regression analysis was employed to determine the prognostic value of this panel. Furthermore, the CIBERSORT and TIMER databases were applied to analyze the correlations among the hub RNAs and immune infiltration, immune checkpoints, and immune cell gene markers in GC.

## 2 Methods and materials

### 2.1 Data acquisition and handling

**FIGURE 1:**
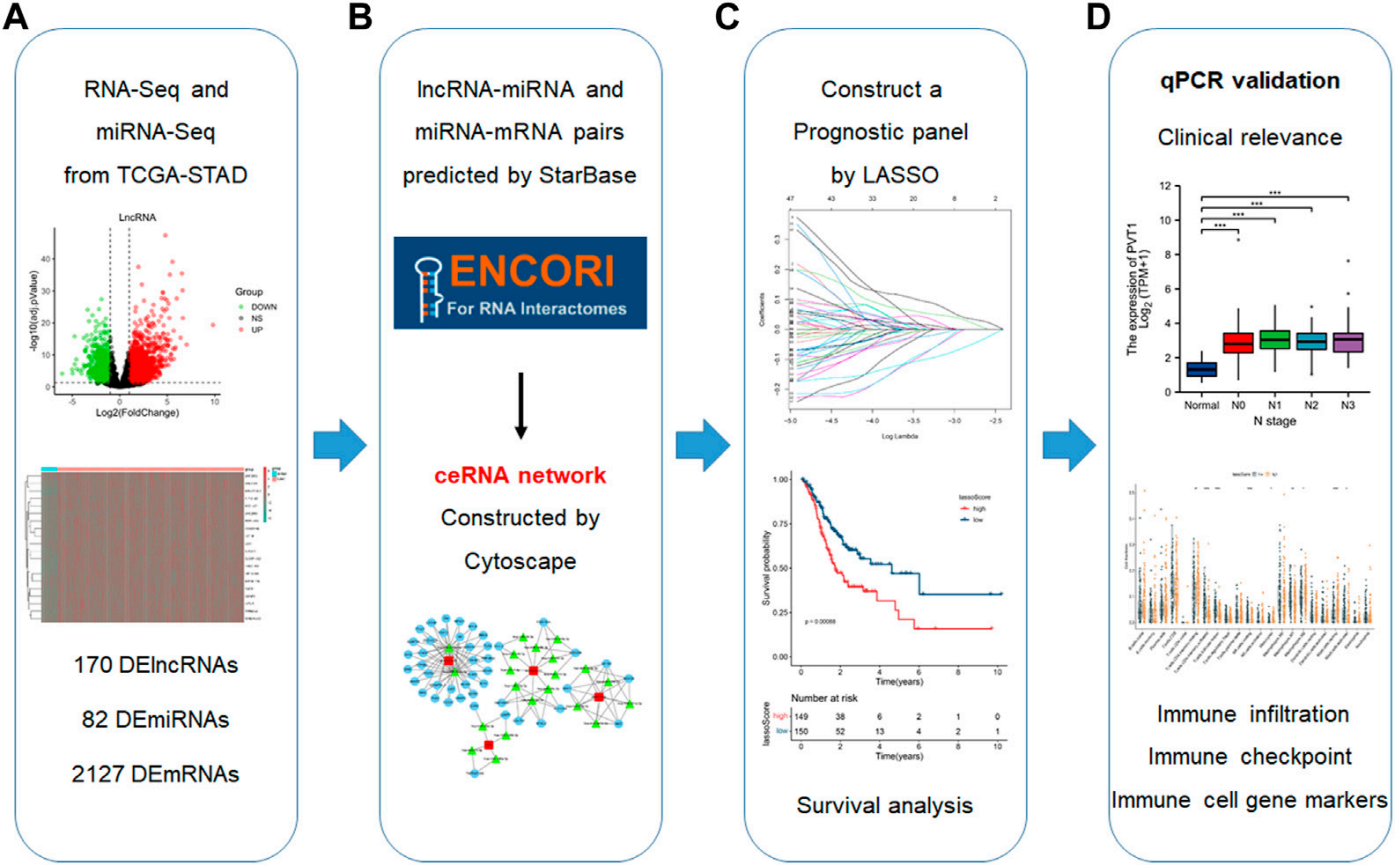
Flowchart for the construction of a prognosis panel based on the ceRNA network and related analyses. **(A)** Data acquisition and handling **(B)** Establish a ceRNA network. **(C)** Construct a prognostic panel by LASSO based on the ceRNA network. **(D)** qPCR validation, analyses of clinical correlations and immune infiltration.

The processing flow of this research is performed in Figure 1. The RNA-seq of 380 cases and the miRNA-seq profiles of 436 cases in gastric cancer and their relative clinical data were acquired from the TCGA database (https://portal.gdc.cancer.gov/). All RNA data were normalized with the “GDCRNAtools” package. The StarBase version 2.0 database (https://starbase.sysu.edu.cn/) was used to transform the miRNA names.

### 2.2 Identification of DElncRNAs, DEmiRNAs and DEmRNAs

The RNA-seq and miRNA-seq profiles were analyzed with the “DESeq” package. We analyzed the different expressed lncRNAs, miRNAs and mRNAs (DElncRNAs, DEmiRNAs and DEmRNAs) in GC using thresholds of foldchange >2 and *p* < 0.01. Volcano maps were generated by the “ggplots” package. Heatmaps were produced by the “gdcHeatmap” package, and the top 20 DElncRNAs, DEmRNAs and DEmiRNAs are shown.

### 2.3 Establishment of the ceRNA network in GC

LncRNAs can function as miRNA sponges and influence mRNA expressions via competitively binding to MRE. The interactions of lncRNA/miRNA pairs and miRNA/mRNA pairs were predicted in the starBase database (Li et al., 2014). A lncRNA‒miRNA-mRNA regulatory ceRNA network was built to scrutinize the regulatory connetion of lncRNA‒miRNA pairs and miRNA‒mRNA pairs in Cytoscape software (version 3.6.1, https://www.cytoscape.org/). The cytoHubba plugin was utilized to find out the top 10 hub elements of the regulatory ceRNA network.

### 2.4 Gene Ontology (GO) enrichment and KEGG pathway analyses

Gene Ontology (GO) enrichment and Kyoto Encyclopedia of Genes and Genomes (KEGG) functional and pathway enrichment analyses were analyzed with the “clusterProfiler” R package and visualized by the “ggplot2” R package.

### 2.5 Construction and prognosis analysis of the LASSO model

LASSO Cox regression of overall survival was employed to estimate the prognostic values of all lncRNAs, miRNAs and mRNAs in the ceRNA network. The “glmnet” R package was adopted to find out the most meaningful prognostic biomarkers. The risk score of GC samples was measured based on the prognosis panel. Hub RNAs identified by the LASSO model based on the ceRNA network were used for further analyses.

### 2.6 Establishment of a nomogram and prediction accuracy evaluation

Base on the risk model build by LASSO, a nomogram comprising clinical characteristic and risk score was established using the R package “survival” and “regplot”. The area under the receiver operating characteristic (ROC) curve (AUC) was adopted to evaluate the accuracy of the nomogram prediction. A calibration plot was performed to assess the assistent between nomogram prediction probability and observation probability.

### 2.7 Survival analysis

The Kaplan‒Meier Plotter database (KM plot, http://kmplot.com) (Lánczky and Győrffy, 2021) is widely deployed to study the correlation between RNA expression and survival in GC. KM plot was employed to describe the association between the expression of hub RNAs and survival time (OS as well as progression-free survival (PFS) in GC.

### 2.8 Clinical sample collection and clinical ethics approval

The sample collection procedure was approved by the Sun Yat-sen University Health Science Institution Review Board (No. KY-2022-051-02). Gastric cancer samples and normal tissues from 32 patients were acquired from the Seventh Affiliated Hospital of Sun Yat-sen University and the First Affiliated Hospital of Sun Yat-sen University. Samples were collected for real-time PCR, immunohistochemistry and Western Blots.

### 2.9 Real-time PCR analysis of hub RNAs

Total RNA was extracted from the tissue samples using AG RNAex Pro RNA reagent (Accurate Biology, CAT#AG21102) according to the manufacturer’s instructions. The cDNA for lncRNA or mRNA qPCR was synthesized using Evo M-MLV reverse transcription master mix (Accurate Biology, CAT# AG11706). miRNA cDNA was synthesized with a miRNA First Strand cDNA Synthesis (Tailing Reaction) kit (Sangon Biotech, B532451-0010). qRT‒PCR was performed with a SYBR Green Pro Tag HS premixed qPCR kit (Accurate Biology, CAT# AG11701). The expression of hub RNAs in the LASSO model was detected by qPCR. The 2^−ΔΔCT^ method was utilized to determine the expression of hub RNAs. LncRNA and mRNA levels were normalized to β-actin, while miRNA expressions were normalized to the expression of U6. The primer sequences of all RNAs used for the qPCR are recorded in Supplementary Table S1.

### 2.10 Correlation of the clinicopathological parameters and the hub RNAs expressions

The RNA-seq and miRNA-seq data in FPKM format were converted into log2. Patients without clinical information were filtered, and patients with sufficient clinical data were retained. We investigated the association between the clinicopathological parameters (T grade, M stage, and N stage) and lncRNA, miRNA and mRNA expression in GC.

### 2.11 In-depth analysis of mRNA RECK in GC

From the LASSO algorithm, we identified a novel prognostic panel consisting of a lncRNA, a miRNA and one mRNA RECK. mRNA can encode proteins and perform biological functions.

The expression of RECK in GC tissue and the OS rate was studied in the KM plot. The OncoPrint diagram of cBioPortal (http://www.cbioportal.org/) shows the distribution of RECK genomic alterations. The relationship between RECK copy number and the mRNA expression level was evaluated by cBioPortal.

### 2.12 Immunohistochemistry (IHC)

Immunohistochemistry was performed to detect RECK expression. Paraffin-embedded tissue sections were deparaffinized and rehydrated in a series of xylene and graded ethanol solutions. Antigen retrieval was performed by boiling the slides in a citrate buffer solution (pH 6.0) for 10 min. Endogenous peroxidase activity was quenched by incubating the slides with 3% hydrogen peroxide for 10 min. After blocking with 5% goat serum for 30 min at room temperature, the sections were incubated with primary antibodies at 4°C overnight. The primary antibody used in this study was [Anti-RECK Rabbit Polyclonal Antibody (ER64778), Huabio, 1:100]. The following day, the sections were washed with phosphate-buffered saline (PBS) and incubated with the corresponding secondary antibody [Goat Anti-Rabbit IgG H&L (HRP) (ab205718), Abcam, 1:1000] for 1 h at room temperature. The sections were then washed with PBS and developed with 3,3′-diaminobenzidine (DAB) solution for 4 min. Counterstaining was performed using hematoxylin for 30 s. Finally, the sections were dehydrated, cleared, and mounted with a cover slip. Positive staining was indicated by brown or yellowish-brown staining in the cytoplasm of the cells. Negative control sections were incubated with normal rabbit serum instead of primary antibody. Slides were observed and images were captured using a microscope.

### 2.13 Western blot (WB)

Further, Western blot was utilized to detect RECK expression. Protein was extracted from the tissue samples using RIPA lysis buffer (Beyotime, P0013B) containing 1% protease inhibitor cocktail (Beyotime, P1005). The protein concentration was determined using a BCA protein assay kit (Thermofisher, 23,225). Equal amounts of protein (20 μg) were separated on a 10% SDS-PAGE gel and then transferred to a polyvinylidene fluoride (PVDF) membrane (Millipore, IPFL00010). The membrane was blocked with 5% milk in Tris-buffered saline with 0.1% Tween-20 (TBST) for 1 h at room temperature and then incubated overnight at 4°C with primary antibodies against the target protein [Anti-RECK Rabbit Polyclonal Antibody (ER64778), Huabio, 1:1000] and GAPDH [GAPDH Monoclonal antibody (60004-1-Ig), Proteintech, 1:20000]. After washing three times with TBST, the membrane was incubated with secondary antibody [Goat Anti-Rabbit IgG H&L (HRP) (ab205718), Abcam, 1:5000] for 1 h at room temperature. The protein bands were visualized using an ECL kit (Beyotime, P0018S) and quantified using ImageJ software. The intensity of the target protein band was normalized to that of the GAPDH band.

### 2.14 Correlation of lassoScore and immune infiltration in GC

CIBERSORT (https://cibersortx.stanford.edu/) was applied to estimate different kinds of tumor-infiltrating immune cells (TIICs) from each sample (Newman et al., 2015), including resting and activated CD4^+^ T cells and naïve B cells. Raw RNA-seq and miRNA data obtained from TCGA were normalized before CIBERSORT analysis. A determined *p*-value (P0.05) was used to assess the deconvolution results’ statistical significance. The relationship between the risk score of the prognostic panel and immune cells was evaluated using Spearman’s correlation test. The expression of the lncRNAs, miRNAs, and mRNAs were estimated to be correlated with the risk score of the prognostic panel using Pearson’s test.

### 2.15 Immune infiltrations of GC

To reveal the association between RECK expression and immune infiltrations, the TIMER database (https://cistrome.shinyapps.io/timer/) was utilized (Li et al., 2020a). The TIMER database is utilized to evaluate the relationship between RECK and immune cell infiltrations (such as T cells, macrophages and B cells et al.), the RECK copy number in GC and their prognostic value. The HPA database was exploited to show RECK expression in different kinds of PBMCs (Uhlen et al., 2019).

Furthermore, the TIMER database was utilized to estimate the correlation of RECK expression with diverse clusters of genetic markers in immune cells (including markers of TAMs, B cells, T cells et al).

### 2.16 Immune checkpoint analysis

The composition of the tumor immune microenvironment (TIME) may affect the response to immune checkpoint blockade (ICB) (Petitprez et al., 2020) which might lead to a poor prognosis. Examining the link between RECK expression and immunological checkpoints (such as PDCD1, CTLA4, CD247, and LAG3) was done using the immunomodulator module of the TISIDB database (http://cis.hku.hk/TISIDB/index.php) (Ru et al., 2019).

### 2.17 Statistical analyses

Statistical analyses were operated with the usage of SPSS (SPSS IBM Corp., Armonk, NY) and R software (version 4.1.1) with the appropriate packages. To compare the variations between the two groups, a *t*-test or comparative *t*-test was employed. Statistical significance was defined as a *p*-value less than 0.05.

## 3 Result

### 3.1 DElncRNAs, DEmiRNAs and DEmRNAs in GC

**FIGURE 2:**
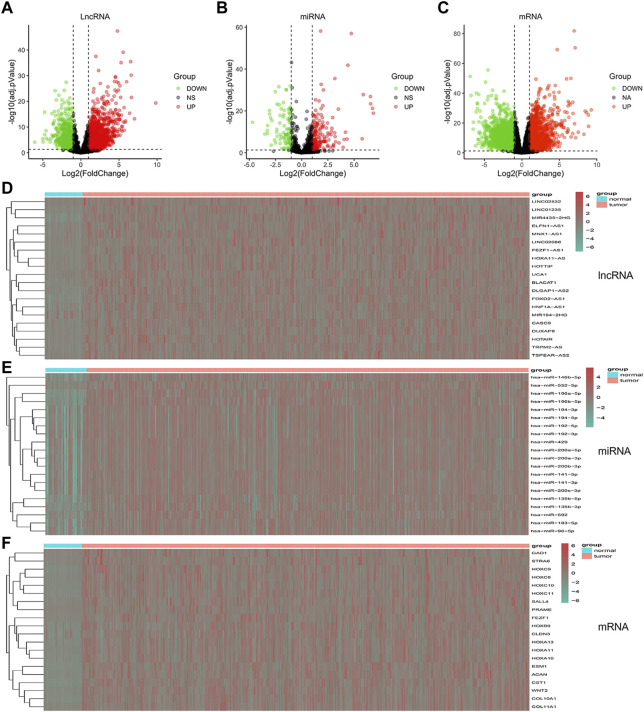
Volcano plots and heatmaps plots of hub RNAs involved in the prognostic panel. **(A–C)** Volcano plots showed DElncRNAs, DEmiRNAs, and DEmRNAs (FoldChange >2 and *p* < 0.01). **(D–F)** Heatmaps showed the top 20 DElncRNAs, DEmiRNAs, and DEmRNAs.

To build a comprehensive ceRNA network, we initially identified DElncRNAs, DEmiRNAs and DEmRNAs with a threshold of log2FC > 2 and *p* < 0.01. In total, 2354 DEmRNAs, 82 DEmiRNAs and 170 DElncRNAs were identified from the TCGA database. Volcano plots were applied to visualize the distribution of DElncRNAs, DEmiRNAs and DEmRNAs (Figures 2A–C). Heatmaps were constructed showing the top 20 significant DEmRNAs, DElncRNAs as well as DEmiRNAs in the GC samples (Figures 2D–F).

### 3.2 Establishment of the ceRNA network

**FIGURE 3:**
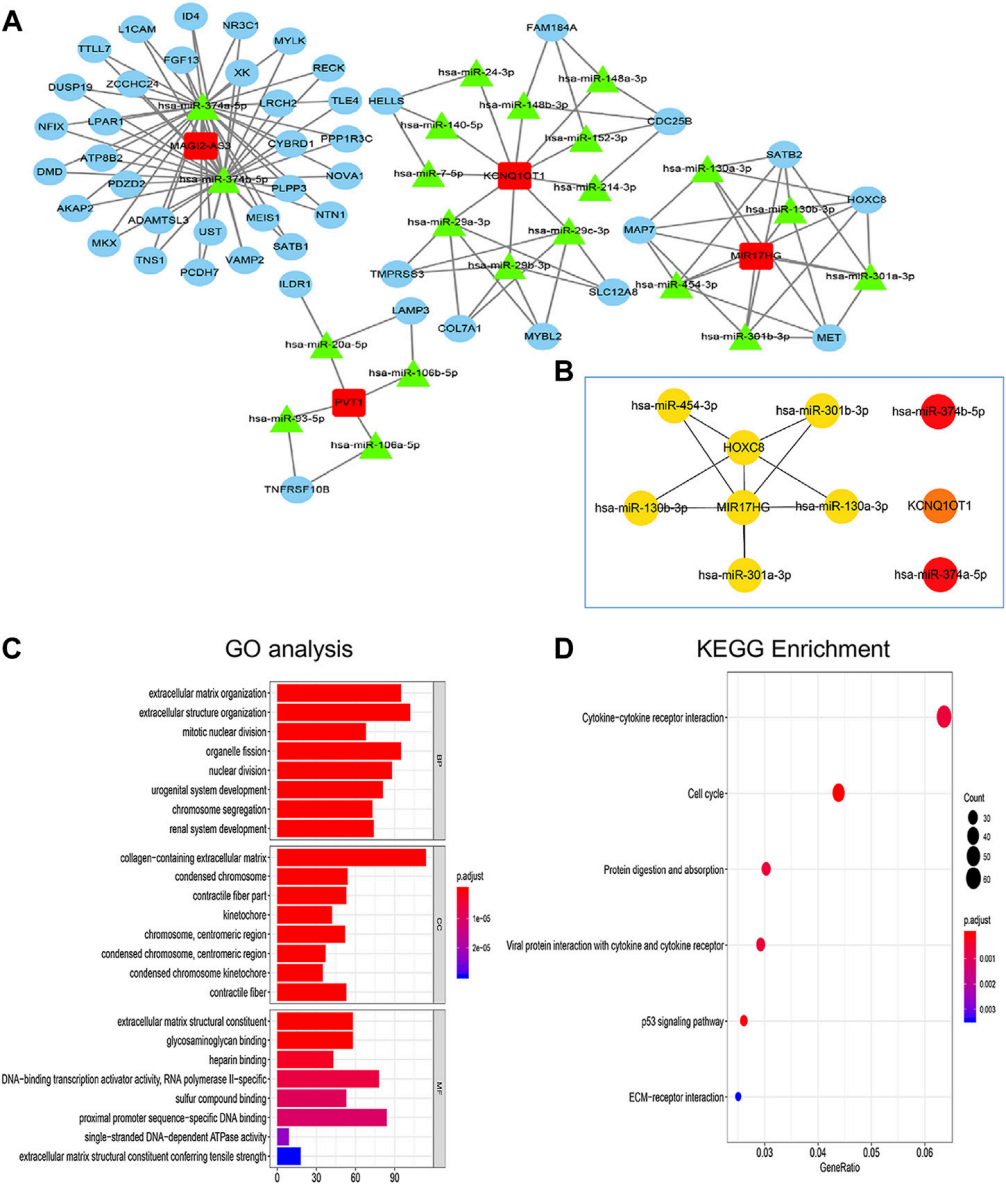
Construction of lncRNA-miRNA-mRNA ceRNA network and functional enrichment analyses. **(A)** The regulatory ceRNA network in gastric cancer. The rectangle represents lncRNA, the triangle represents miRNA and the circle represents mRNA. **(B)** The top ten hub RNAs of the ceRNA network were visualized by the CytoHubba plug-in of Cytoscape. **(C,D)** Functional enrichment analyses (including GO and KEGG).

The lncRNAs‒miRNAs and miRNAs‒mRNAs interactions were explored in the starBase database to establish the triple ceRNA network for gastric cancer. Then, 4 lncRNAs, 21 microRNAs and 45 mRNAs were incorporated into the ceRNA network with the usage of Cytoscape software (Figure 3A).

The hub elements of the GC regulating ceRNA network were identified using the CytoHubba plug-in in Cytoscape. One lncRNA (MIR17HG), seven miRNAs and two mRNAs (HOXC8 and KCNQ1OT1) were identified (Figure 3B). GO analysis was employed to assess the functions of the DEmRNAs. Biological process (BP) enrichment indicated that the DEmRNAs were particularly enriched in the extracellular matrix organization, cellular component (CC) enriched in the collagen−containing extracellular matrix, the extracellular matrix organization along with MF enriched in extracellular matrix structural constituent (Figure 3C). According to KEGG analysis, the DEmRNAs were connected to cytokine‒cytokine receptor interactions, the cell cycle function, protein digestion and absorption, viral protein interactions with cytokines and cytokine receptors, ECM-receptor interactions and the p53 signaling pathway (Figure 3D).

### 3.3 Construction of a prognostic panel using a LASSO algorithm

**FIGURE 4:**
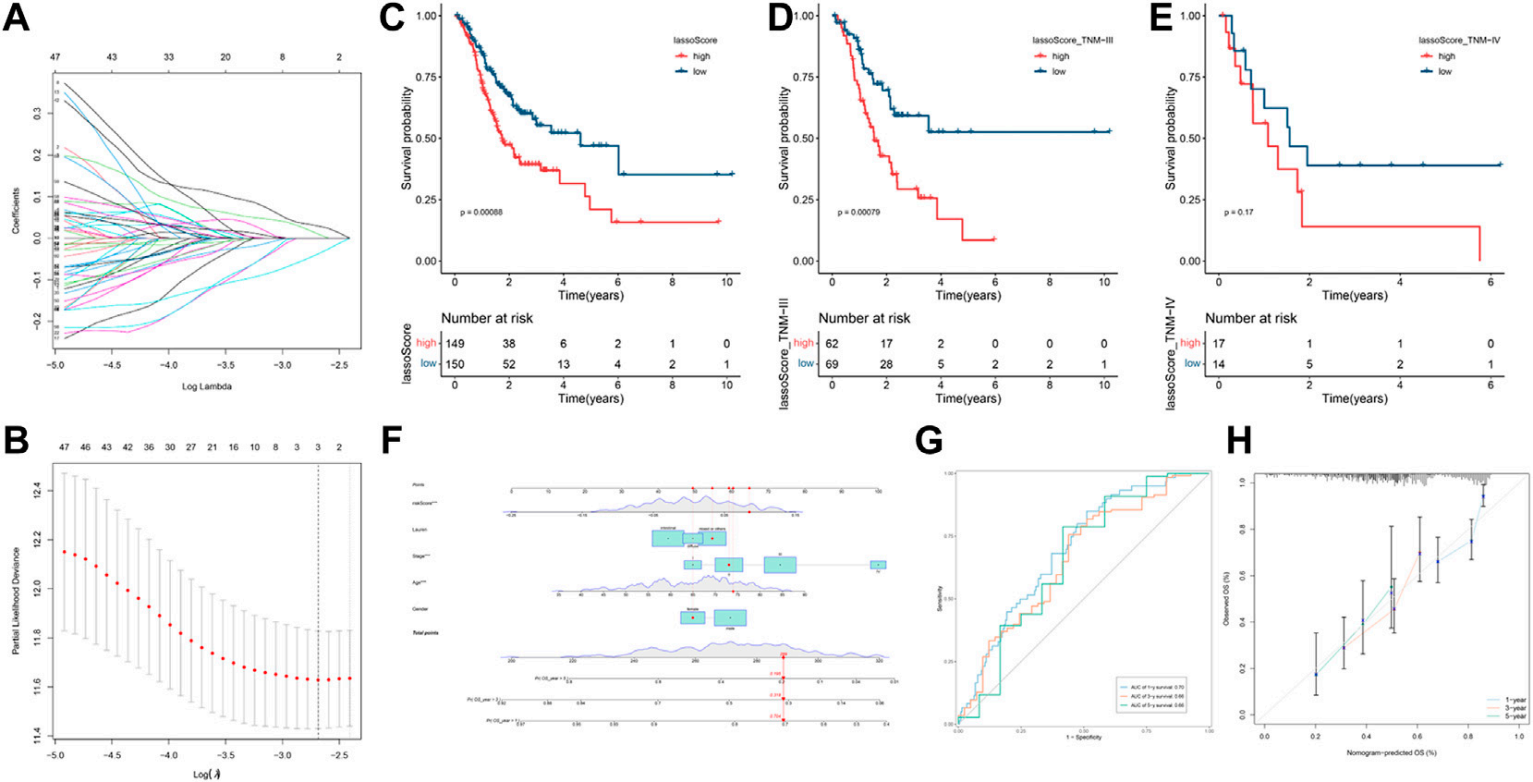
Construction and prognosis analysis of the LASSO model. **(A)** LASSO coefficient profiles. **(B)** Ten cross-validations utilized to select suitable parameters. **(C–E)** Kaplan-Meier curve is utilized to evaluate the overall survival (OS) between high lassoScore and low lassoScore groups in GC. **(F)** Nomogram model to predict the risk of OS base on the LASSO model and clinical features. Note: five factors (including riskScore, Lauren classification, Stage, Age and Gender) were included in the Nomogram model. **(G)** The area under the receiver operating characteristic (ROC) curve (AUC) was used to evaluate the accuracy of the nomogram prediction. **(H)** Calibration curve was used to analyze the predictive accuracy of the nomogram.

The LASSO algorithm was employed to establish a novel prognostic panel of GC based on the regulatory ceRNA network. LASSO Cox regression with 10-fold cross-validation (Figures 4A, B) for the clinical prognostic effect of gastric cancer identified one lncRNA (PVT1), one miRNA (hsa-miR-130a-3p) and one mRNA (RECK). The lncRNA‒miRNA-mRNA expression risk score was developed using the following formula: −0.0377165916846377× lncRNA PVT1 +0.0102114413809827 × hsa-miR-130a -3p + 0.0295683968712249 × RECK. The STAD-TCGA patients with elevated lassoScore demonstrated a noticeably poorer survival than those with low lassoScore, according to Kaplan-Meier analysis (Figure 4C, *p* = 0.00088; Figure 4D, *p* = 0.00079).

### 3.4 Construction and evaluation of the nomogram for predicting prognosis

We constructed a nomogram to evaluate the survival of gastric cancer. Nomogram based on the Lasso riskScore and clinical features (including Lauren classification, Stage, Age and Gender). It can be clearly seen from the nomogram that the total score of this patient was 289 points and the corresponding probability of 1-year, 3-year, and 5-year survival rate was 0.195, 0.318, 0.704 respectively (Figure 4F). We analyzed the accuracy of the nomogram for gastric cancer prognosis by ROC analysis (Figure 4G). Areas under the curve (AUC) were 0.70, 0.66, and 0.66 for 1-year, 3-year, and 5-year, and respectively.

As shown by the calibration curve, it can be seen that the model performed well in predicting the 3-year and 5-year survival probabilities, but showed some errors in predicting the 1-year survival probability (Figure 4H). This indicates that the predictive accuracy of this model is higher in the shorter time period, but needs further improvement for short-term predictions.

### 3.5 qPCR validation and clinical relevance of lncRNA PVT1, hsa-miR-130a-3p and mRNA RECK

**FIGURE 5:**
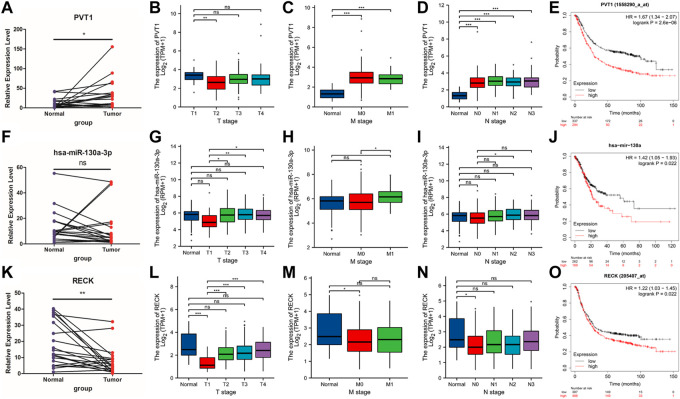
qPCR validation and clinicopathological parameters of PVT1, hsa-miR-130a-3p and RECK. **(A,F,K)** The expression of PVT1, hsa-miR-130a-3p and RECK between the tumor group and the normal group **(B–D)**; **(G–I)**; **(L–N)**. **(E)** KM plot was utilized to describe the OS in the high or low PVT expression groups. **(J)** KM plot was used to show the OS in the high or low hsa-mir-130a expression groups. **(O)** KM plot was applied to present the OS in the high or low RECK expression groups.

Real-time PCR was used to evaluate the hub RNAs implicated in the LASSO model in clinical GC samples as well as normal control samples. In comparison to the normal group, the tumor group’s expression of PVT1 was considerably higher (Figure 5A, *p* = 0.0096 < 0.05). The expression of hsa-miR-130a-3p was significantly upregulated in the tumor than normal groups (Figure 5F, *p* = 0.0349 < 0.05). The expression of RECK declined in the tumor group compared to the normal group (Figure 5K, *p* = 0.0040 < 0.05).

Furthermore, we scrutinized the associations between hub RNAs expression (including PVT1, hsa-miR-130a-3p and RECK) and the TMN stage. The results figured out that high lncRNA PVT1 expression was positively associated with T, M, and N stage (Figures 5B–D), while hsa-miR-130a-3p expression was not significantly correlated with T stage, M stage or N stage (Figures 5G–I). The mRNA RECK expression was significantly decreased in the T1 stage, M0 stage and N0 stage compared with the normal group, while no significant differences were found between any other groups (Figures 5L–N).

Next, the Kaplan‒Meier plotter was administered to independently estimate the prognostic value of PVT1, hsa-miR-130a-3p, and RECK expression in GC. High PVT1 expression was related to a poor prognosis in GC (Figure 5E, *p* = 2.6 × 10^−6^). High expression of hsa-miR-130a-3p was statistically significantly linked to poor OS in GC patients (Figure 5J, *p* = 0.022). High expression of RECK was related to poor OS in GC patients (Figure 5O, *p* = 0.022).

The relationship between the expression of PVT1, hesa-miR-130a-3p and RECK and the prognosis of clinical-pathological features of gastric cancer was further analyzed. The findings demonstrated that GC patients with various clinical-pathological characteristics, including males and females, those undergoing surgery or 5-FU-based adjuvant therapy, and those who were HER2-negative or HER2-positive, had a worse prognosis when high PVT1 expression was present (Table 1, *p* < 0.05). High expression of hsa-miR-130a-3p was associated with poor prognosis in male patients, those with stage 2 or grade 2 disease, those with high mutation burden, those with CD4^+^ memory T-cells enriched, CD8^+^ T-cells decreased, macrophages decreased, B-cells decreased, eosinophils decreased, and natural killer T-cells enriched (Table 2, *p* < 0.05). Furthermore, in gastric cancer patients with a variety of clinicopathological features, such as T stage 2 or 4 or N+ stage, M0 stage, surgery, 5-FU-based adjuvant therapy, other adjuvant therapy, and negative HER2, increased RECK expression was linked to a worse prognosis (Table 3, *p* < 0.05).

**TABLE 1:** Correlation of PVT1 expression and clinical prognosis in gastric cancer regarding various clinicopathological factors.

Clinicopathological characteristics	OS (*n* = 875)			FPS (*n* = 640)		
	N	Hazard ratio	*p*-value	N	Hazard ratio	*p*-value
Sex						
Female	236	1.75 (1.05–2.93)	0.029^a^	201	2.48 (1.69–3.63)	1.5*10^-6^a^
Male	544	1.82 (1.36–2.45)	5.5*10^-5^a^	437	1.85 (1.43–2.38)	1.5*10^-6^a^
T Stage						
1	-	-	-	-	-	-
2	241	1.88 (1.09–3.23)	0.021^a^	239	0.8 (0.53–1.22)	0.3
3	204	1.73 (1.22–2.44)	0.0017^a^	204	1.13 (0.78–1.65)	0.51
4	38	3.53 (1.4–8.91)	0.0047^a^	39	1.59 (0.71–3.56)	0.26
N Stage						
N0	74	2.23 (0.96–5.17)	0.055	72	1.9 (0.64–5.6)	0.24
N+	422	1.56 (1.2–2.03)	0.00091^a^	423	1.18 (0.9–1.53)	0.23
M Stage						
M0	444	1.78 (1.34–2.35)	4.2*10^-5^a^	443	1.3 (0.99–1.72)	0.061
M1	56	0.72 (0.4–1.28)	0.26	56	0.66 (0.34–1.26)	0.2
Differentiation						
Poorly	165	2.16 (1.3–3.59)	0.0024^a^	121	0.76 (0.49–1.2)	0.25
Moderately	67	2.49 (1.17–5.3)	0.015^a^	67	0.68 (0.34–1.34)	0.26
Well	32	2.86 (0.83–9.8)	0.081	-	-	-
Treatment						
Surgery	380	1.47 (1.09–1.97)	0.011^a^	375	1.38 (1.03–1.84)	0.028^a^
5-FU based adjuvant	152	2.29 (1.6–3.29)	4.2*10^-6^a^	152	2.32 (1.6–3.35)	4.4*10^-6^a^
others adjuvant	76	1.55 (0.62–3.9)	0.34	80	1.76 (0.78–3.97)	0.17
HER2						
Negative	532	1.66 (1.32–2.08)	1.1*10^-5^a^	408	1.68 (1.28–2.21)	0.00019^a^
Positive	343	1.55 (1.18–2.01)	0.0012^a^	232	1.94 (1.4–2.68)	4.5*10^-5^a^

^a^ Means *p* < 0.05.

**TABLE 2:** Correlation of hsa-miR-130a expression and clinical prognosis in GC concerning various clinicopathological factors and immune cells.

Clinicopathological characteristics	OS (*n* = 7,642)		
	N	Hazard ratio	*p*-value
Sex			
Female	154	1.4 (0.83–2.37)	0.21
Male	277	1.84 (1.13–2.98)	0.013^a^
Stage			
1	55	1.85 (0.59–5.75)	0.28
2	128	2.12 (1.06–4.22)	0.029^a^
3	178	1.45 (0.94–2.23)	0.09
4	43	1.84 (0.69–4.95)	0.22
Grade			
1	-	-	-
2	153	2.16 (1.26–3.71)	0.004^a^
3	259	0.81 (0.55–1.2)	0.29
4	-	-	-
Mutation burden			
High	215	2.04 (1.12–3.72)	0.017^a^
Low	210	1.45 (0.96–2.19)	0.075
Neoantigen load			
High	73	2.7 (0.95–7.67)	0.052
Low	-	-	-
*Restrict analysis based on cellular content*			
CD4^+^ memory T-cells			
Enriched	254	1.83 (1.07–3.15)	0.026^a^
Decreased	152	1.44 (0.84–2.45)	0.18
CD8^+^ T-cells			
Enriched	204	1.51 (0.9–2.55)	0.21
Decreased	202	2.05 (1.28–3.28)	0.0023^a^
Macrophages			
Enriched	217	1.46 (0.86–2.49)	0.16
Decreased	189	2.05 (1.28–3.29)	0.0023^a^
B-cells			
Enriched	229	1.37 (0.9–2.08)	0.14
Decreased	177	2.3 (1.23–4.31)	0.0076^a^
Eosinophils			
Enriched	304	1.74 (1.21–2.5)	0.0024^a^
Decreased	112	1.99 (0.95–4.19)	0.064
Natural killer T-cells			
Enriched	260	1.61 (1.08–2.41)	0.018^a^
Decreased	146	1.37 (0.83–2.28)	0.22

^a^ Means *p* < 0.05.

**TABLE 3:** Correlation of RECK expression and clinical prognosis in gastric cancer with various clinicopathological factors.

Clinicopathological characteristics	OS (*n* = 875)			FPS (*n* = 640)		
	N	Hazard ratio	*p*-value	N	Hazard ratio	*p*-value
Sex						
Female	236	1.66 (1.14–2.42)	0.0079^a^	201	1.5 (0.99–2.27)	0.054
Male	544	1.22 (0.96–1.56)	0.098	437	1.19 (0.92–1.54)	0.18
T Stage						
1	-	-	-	-	-	-
2	241	1.8 (1.18–2.75)	0.0058^a^	239	2.03 (1.17–3.54)	0.01^a^
3	204	1.26 (0.91–1.83)	0.15	204	1.26 (0.91–1.77)	0.17
4	38	3.36 (1.36–8.28)	0.0053^a^	39	2.65 (1.17–5.99)	0.015^a^
N Stage						
N0	74	2.29 (0.92–5.68)	0.066	72	2.19 (0.88–5.41)	0.083
N+	422	2.06 (1.57–2.69)	7.9*10^-8^a^	423	1.86 (1.44–2.41)	1.7*10^-6^a^
M Stage						
M0	444	1.8 (1.36–2.38)	3.2*10^-5^a^	443	1.6 (1.22–2.1)	0.00055^a^
M1	56	2.13 (1.17–3.88)	0.011^a^	56	1.68 (0.92–3.08)	0.088
Differentiation						
Poorly	165	0.79 (00.5–1.25)	0.31	121	1.19 (00.75–1.87)	0.46
Moderately	67	1.43 (0.74–2.77)	0.28	67	1.42 (0.74–2.75)	0.29
Well	32	2.39 (0.87–6.54)	0.081	-	-	-
Treatment						
Surgery	393	158 (1.18–2.13)	0.0022^a^	375	1.52 (1.1–2.11)	0.0099^a^
5-FU based adjuvant	152	0.62 (0.42–0.91)	0.013^a^	152	0.58 (0.39–0.84)	0.0041^a^
others adjuvant	76	3.38 (1.35–8.5)	0.0059^a^	80	2.96 (1.33–6.62)	0.0055^a^
HER2						
Negative	532	1.42 (1.12–1.8)	0.0037^a^	408	1.42 (1.08–1.85)	0.01^a^
Positive	343	1.52 (1.15–2)	0.0028^a^	232	1.2 (0.85–1.69)	0.3

^a^ Means *p* < 0.05.

### 3.6 Expression, mutation and prognostic value of RECK

**FIGURE 6:**
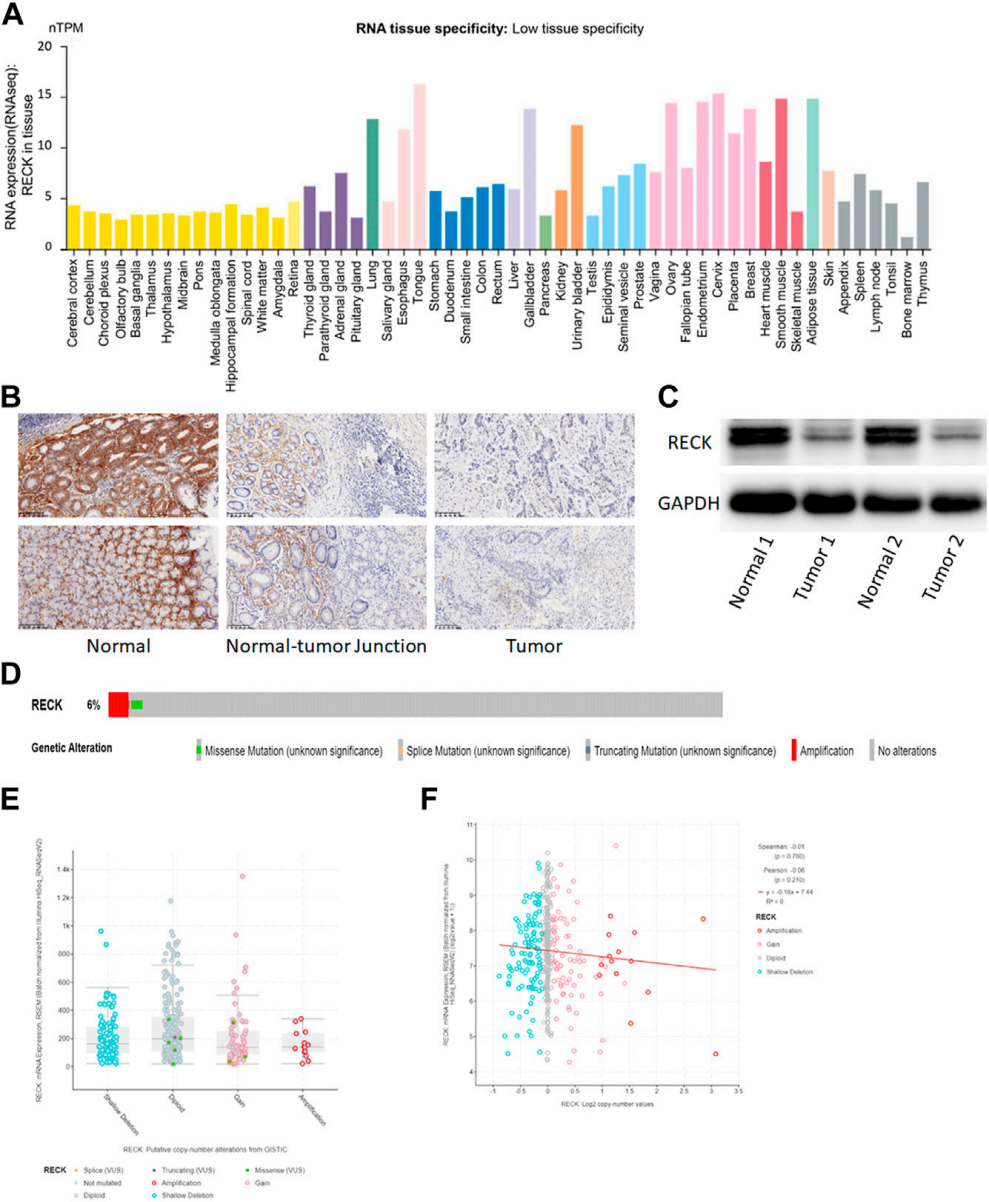
Expression, mutation and prognostic value of RECK. **(A)** Distribution of RECK expression in pan-cancerous tissues. **(B)** Immunohistochemistry analysis showed that the expression of RECK in gastric cancer was lower than the normal tissue (200x). **(C)** Western blot showed that the expression of RECK in gastric cancer was lower than the normal tissue. **(D)** The distribution of RECK genomic alterations in TCGA-STAD is illustrated using cBioPortal OncoPrint plots. **(E,F)** The relationship between RECK copy number and mRNA expression was visualized with dot plots **(E)** and correlation plots **(F)** using cBioPortal.

The HPA database was utilized to describe the expression of RECK in pan-cancer tissue (Figure 6A). Immunohistochemistry (IHC) staining of clinical gastric cancer sample and normal tissue further confirmed the low expression of RECK in GC tissue compared to the normal group (Figure 6B). Besides, Western blot showed the low expression of RECK in gastric cancer than normal group (Figure 6C). Furthermore, data collected from cBioPortal showed a 6% deletion of the RECK gene in TCGA gastric cancer cases (Figure 6D). Gastric cancer samples harboring shallow deletions showed no differential expression of RECK in comparison to diploid, amplification or gain samples (Figure 6E). PTEN copy number value and mRNA expression were not significantly correlated in GC samples (Figure 6F).

### 3.7 Correlation analysis of the lassoScore and immune infiltrations in gastric cancer

**FIGURE 7:**
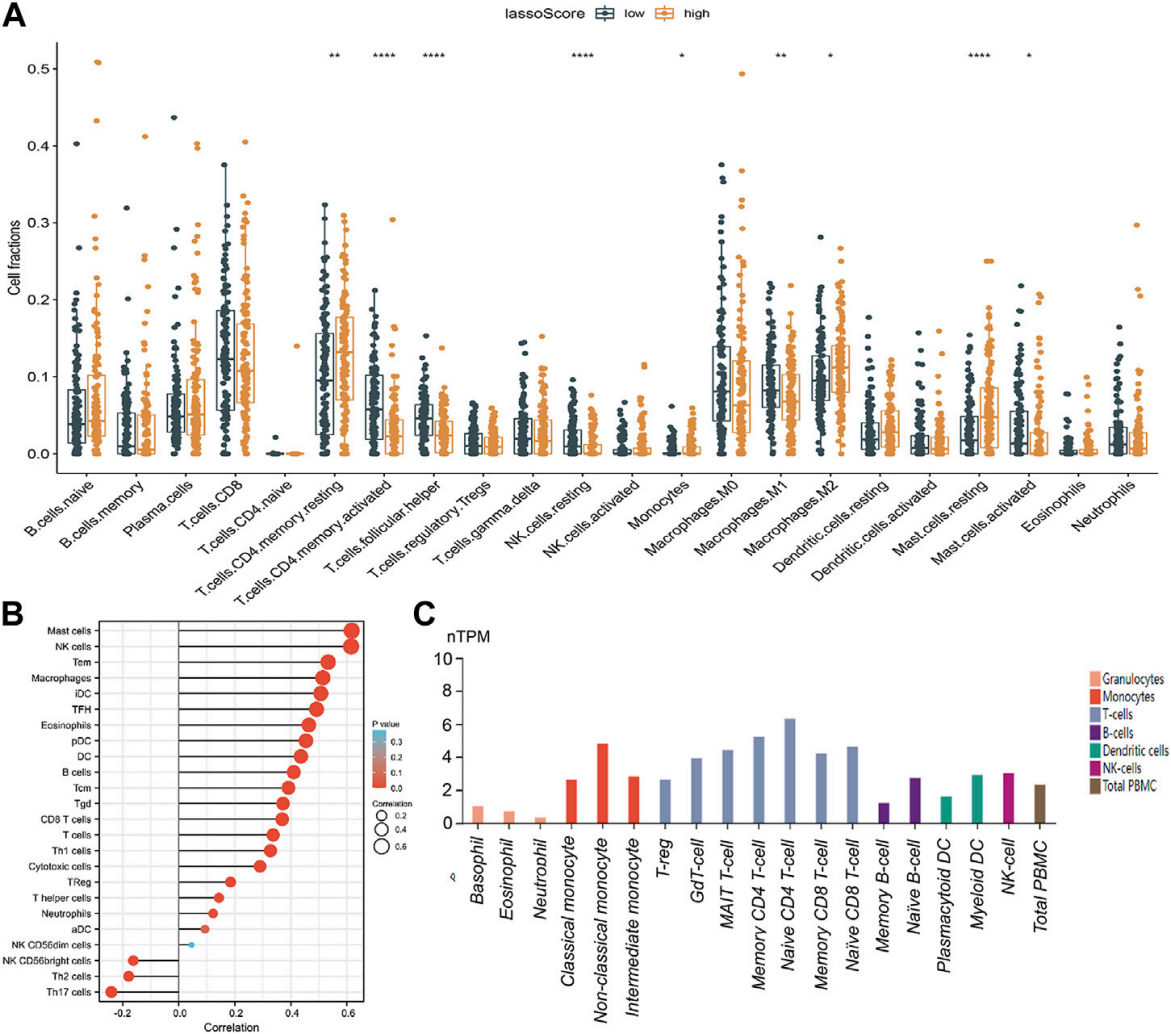
Correlation analysis of the lassoScore or RECK expression and immune infiltration in gastric cancer. **(A)** CIBERSORT analysis estimated the relative ratio of tumor-infiltrating cells in the low lassoScore and high lassoScore groups. **(B)** The relation between RECK expression and immune infiltrations in GC was investigated. **(C)** Expression of RECK in PBMCs from the HPA database.

We carefully examined the correlation between the lassoScore and the 22 different TIICs of gastric cancer from the CIBERSORT in order to ascertain whether this unique ceRNA-based prognostic panel was associated with tumor immunity. Compared with the low lassoScore group, the high lassoScore group exhibited a significantly high ratio of resting memory CD4^+^ T cells (*p* < 0.01), M2 macrophages (*p* < 0.05) and resting mast cells (*p* < 0.0001) and a considerably reduced ratio of activated memory CD4^+^ T cells (*p* < 0.0001), helper follicular T cells (*p* < 0.05), resting natural killer (NK) cells (*p* < 0.0001), M1 macrophages (*p* < 0.01) and activated mast cells (*p* < 0.05) (Figure 7A). Immune infiltration results in this study indicated that this triple panel may indicate alterations in the TIME.

### 3.8 Correlations of RECK expression and immune infiltrations

RECK was the only mRNA included in the prognostic panel constructed by the LASSO algorithm based on the ceRNA network. mRNA can perform biological functions through translation into proteins. To show the connection between RECK expression and immune cell invasion, the TIMER database was used. The outcomes indicated that the expression of RECK was significantly correlated with CD8 T cells, mast cells, NK cells, TEMs, macrophages and B cells (Figure 7B). Furthermore, the HPA database revealed that RECK was expressed in monocytes, naïve and memory CD4 T cells, naive and memory CD8 T cells, T-regs, naïve and memory B cells, myeloid DCs and NK cells (Figure 7C).

**FIGURE 8:**
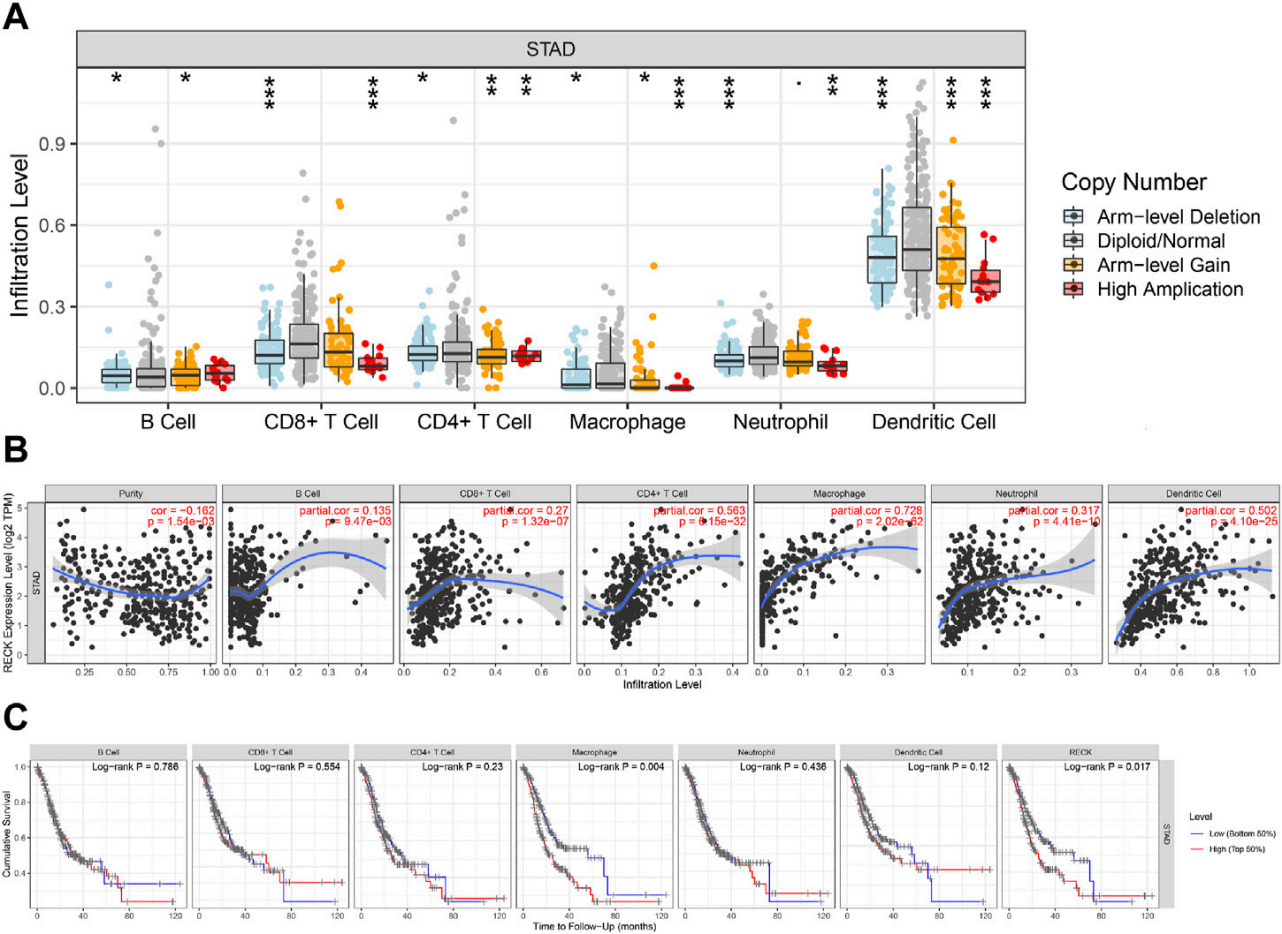
Correlation analyses of RECK expression and the immune infiltrations of GC. **(A)** Relationship between RECK copy number and immune cell infiltration in gastric cancer. **(B)** Correlation of RECK expression with the level of immune infiltrations of GC. **(C)** Kaplan-Meier plots were employed to demonstrate the immune infiltration and OS of GC. Notes: * represents *p* less than 0.05, ** represents *p* less than 0.01, *** represents *p* less than 0.001. Abbreviation: GC: gastric cancer; OS: overall survival.

“SCNA” module analysis in TIMER showed that RECK gene copy numbers were closely related to B cells, CD8^+^ T cells, macrophages, dendritic cells, neutrophils, and CD4^+^ T cells (Figure 8A). Furthermore, the “Gene” module analysis revealed that the mRNA RECK level was remarkably relevant to tumor purity, B cells, CD4^+^ T cells, dendritic cells, macrophages, CD8^+^ T cells and neutrophils (Figure 8B). Finally, the predictive significance of immune infiltration in GC patients was investigated. The results showed that GC patients had a worse prognosis when their levels of macrophages and RECK were higher (Figure 8C, *p* = 0.004, *p* = 0.017, respectively).

To further elucidate the association between RECK and infiltrating immune cells, the TIMER database was utilized. Amazingly, the outcomes reveal a markedly positive correlation between RECK expression and M2 gene markers (VSIG4 and CD163), M1 gene markers (PTGS2 and IRF5), TAM gene markers (IL10, CCL2, and CD68), B-cell gene markers (CD19, CD79A), T-cell gene markers (CD3E, CD3D, and CD2), CD8^+^ T-cell gene markers (CD8A, CD8B), monocyte gene markers (CD86, CSF1R) and neutrophil gene markers (MS4A4A, ITGAM) (Table 4).

**TABLE 4:** Correlation analysis between RECK and gene markers of immune cells in TIMER.

Description	Gene markers	Cor	*p*
TAM	CCL2	0.546	***
	CD68	0.252	***
	IL10	5.54	***
M1	IRF5	0.291	***
	PTGS2	0.268	***
M2	CD163	0.523	***
	VSIG4	0.496	***
B cell	CD19	0.402	***
	CD79A	0.406	***
T cell	CD3D	0.307	***
	CD3E	0.345	***
	CD2	0.381	***
CD8^+^ T cell	CD8A	0.345	***
	CD8B	0.261	***
Monocyte	CD86	0.468	***
	CSF1R	0.613	***
Neutrophils	MS4A4A	0.574	***
	CEACAM8	0.086	0.08
	ITGAM	0.544	***

Notes: * represents *p* less than 0.05, ** represents *p* less than 0.01, and *** represents *p* less than 0.001.

In summary, these findings indicate that the RECK expression identified by the LASSO algorithm was significantly positively related to immune infiltration.

### 3.9 Immune checkpoint analysis

**FIGURE 9:**
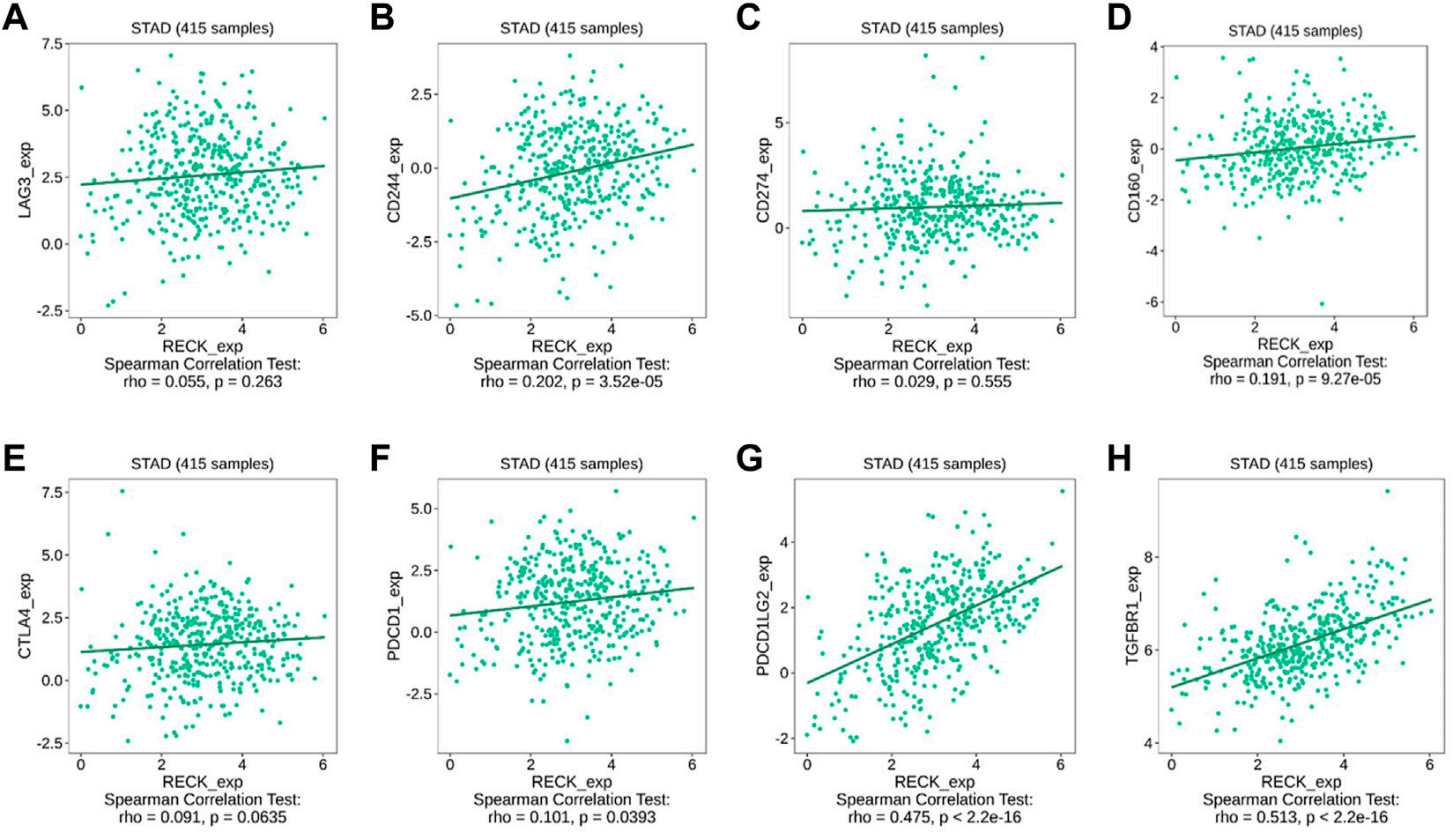
Correlation between RECK and immunological checkpoints **(A–H)**.

From the result above, we pointed out a positive association between RECK expression and immune infiltrations. Earlier research has manifested that the infiltration of tumor-related immune cells is associated with relevant immune checkpoints. The immunomodulator module involved in the TISIDB database was employed to investigate the interrelationship between RECK expression and immune checkpoints, such as LAG3, CTLA4, and PDCD1. The outcomes displayed that the expression of RECK was remarkably positively correlated with CD244 (r = 0.202, *p* = 3.52 × 10^−5^), CD160 (*r* = 0.191, *p* = 9.27 × 10^−5^), PDCD1 (PD1, *r* = 0.101, *p* = 0.0393), PDCD1LG2 (*r* = 0.475, *p* = 2.2 × 10–16), and TGFBR1 (*r* = 0.513, *p* = 2.2 × 10–16) (Figure 9).

## 4 Discussion

Gastric cancer is an age-related disease of which the incidence and mortality rates rise with age (Yang et al., 2018a). It is the third leading cause of cancer-related mortality globally, with a poor clinical prognosis and a frequent occurrence of metastasis. In individuals over 40 and 50 years old, the occurrence and mortality of gastric cancer are significantly higher in males than in females (Yang et al., 2018a). Although chemotherapy and surgical treatment have been extensively used, the outcome of GC is still unsatisfactory. It is meaningful to elucidate the pathogenesis and discover novel biomarkers of gastric cancer.

Aging and cancer share some of the same characteristics, including epigenetic alterations, genomic instability, chronic inflammation and dysbiosis et al. (López-Otín et al., 2023b). Various researches have revealed that ceRNAs (including lncRNAs, circRNAs, miRNAs and mRNAs) play a crucial role in GC tumorigenesis and metastasis (Yuan et al., 2020). Nevertheless, limited research has been conducted on establishing a prognostic panel utilizing a comprehensive ceRNA network in GC.

In the present research, RNA seq and miRNA seq data were obtained from the STAD-TCGA and the DElncRNAs, DEmRNAs and DEmiRNAs were analysed. Second, a ceRNA network was built using the different expression RNAs. Third, a LASSO model was utilized to establish a novel prognostic panel using the ceRNA network of GC. Interestingly, the results of the LASSO model consisted of three hub RNAs, including one lncRNA PVT1, one miRNA hsa-miR-130a-3p and one mRNA RECK. Further study showed that this three-element panel was firmly associated with immune cell infiltration.

lncRNAs, miRNAs and mRNAs influence the progression of GC at different levels directly and indirectly. Intensive studies combining lncRNAs, miRNAs and mRNAs are meaningful to further explore the GC mechanism. In our study, LASSO was utilized to discover the most essential RNA elements. Interestingly, a three-element panel consisting of PVT1, hsa-miR-130a-3p and RECK was built. Individuals in high lassoScore group possessed a worse clinical prognosis than those with the low lassoScore group. This three-element panel (including one lncRNA, one miRNA and one mRNA) based on the ceRNA network might be a new clinical prognostic panel for GC.

PVT1, a well-known oncogenic lncRNA, is usually coexpressed with MYC (MYC proto-oncogene) and is vital for increasing the expression of MYC in cancer (Tseng et al., 2014). PVT1 is implicated in the process of adaptation to hypoxia. Hypoxia-induced PVT1 expression can enhance cisplatin chemoresistance in lung cancer by autophagy through the PVT1/miR-140-3p/ATG5 pathway (Wang et al., 2022). PVT1 stimulated cell proliferation by activating KAT2A acetyltransferase and stabilizing HIF-1, both of which are involved in hypoxia-related regulatory processes (Wang et al., 2020). LncRNA PVT1 is crucial for the development of GC through the ceRNA network. Consistent with a previous study (Li et al., 2020b), our study discovered that the expression of PVT1 was remarkably elevated in the GC group compared to the normal group and high PVT1 expression is related to a worse prognosis. PVT1 improves the migration of gastric cancer cells by competing for binding with miR-30a and influencing the expression of Snail (Wang et al., 2021). LncRNA PVT1 has been identified in gastric juices and is believed to be a prognostic biomarker of gastric cancer (Yuan et al., 2020). LncRNA PVT1 in plasma/serum can function as a diagnostic and prognostic biomarker of GC (Fattahi et al., 2020).

Numerous types of research have disclosed that miRNAs are vital in the regulation of cancer-related genes. miRNAs mainly regulate the function of mRNA by combining with MRE which subsequently led to mRNA degradation. Phosphatase and tensin homolog (PTEN) functions as tumor inhibitor by inhibiting PI3K signaling. miRNA-221/222 has been demonstrated to modulate the expression of PTEN, which subsequently blocks the GC invasion and proliferation. PTEN is also a target binding mRNA of miRNA-21, which promotes GC cell invasion and proliferation. MiRNA-143 regulates the function of GC via the PI3K/Akt pathway (Takagi et al., 2009). Our study disclosed that high expression of hsa-miR-130a-3p was relevant to a worse clinical prognosis. A previous study concluded that miR-130a-3p promoted GC cell growth by targeting GCNT4 and then facilitating the TGF-1/SMAD3 signaling pathway, which was in line with our findings (Hu et al., 2021).

mRNA RECK was included in our study’s three-element prognostic model constructed by the LASSO model. Many mRNAs have been elucidated to be involved in gastric cancer. RECK is a crucial mRNA involved in the development of GC. Our study proved that RECK was decreased in the GC group compared with the normal group and high expression of RECK related to a worse clinical prognosis. miR-590-5p stimulated GC cell proliferation and decreased chemosensitivity via RECK and the AKT/ERK pathway (Shen et al., 2016). MiR-25 improved the growth and motility of GC cells by targeting RECK (Zhao et al., 2014). MiR-374b-5p inhibits the expression of RECK and promotes GC cell metastasis and invasion (Xie et al., 2014). In summary, RECK and its regulation by miRNAs are essential during the development of GC.

According to KEGG enrichment analysis in this study, the DEmRNAs in GC are involved in cytokine‒cytokine receptor interaction, cell cycle, protein digestion and absorption, viral protein interaction with cytokine and cytokine receptor, p53 signaling pathway and ECM-receptor interaction. Researchers have found that inflammatory cells and immune cells can interact through cytokines (including interleukins, interferon, TNF-α, and TGF-β) (Akdis et al., 2016). Consistent with our findings, earlier research demonstrated that the p53 signaling pathway has participated in tumorigenesis, proliferation, and invasion (Huang et al., 2017; Du et al., 2018).

The TIME is crucial in tumorigenesis and development (Binnewies et al., 2018). Immune cells in the TIME possess tumor-antagonistic or tumor-promoting characteristics (Lei et al., 2020). There are two categories of tumor-associated immune cells in TIME according to their functions in cancer, including tumor-antagonistic immune cells (TAICs) and tumor-promoting immune cells (TPICs) (Pansy et al., 2021). TAICs primarily comprise dendritic cells (DCs), M1-polarized macrophages, NK cells, effector T cells, and N1-polarized neutrophils (Akdis et al., 2016). TPICs chiefly comprise regulatory T cells (Tregs) and myeloid-derived suppressor cells. Tumor-related immune cells improve tumor development by secreting cytokines and promoting metastasis through the production of growth factors and matrix-degrading enzymes (Bi et al., 2022). Immune infiltration could also influence the clinical prognosis. In our study, we demonstrated that a high lassoScore was significantly associated with a high ratio of M2 macrophages, resting memory CD4^+^ T cells and resting mast cells. Further, our study showed that the expression of RECK was significantly correlated with CD8 T cells, mast cells, NK cells, TEMs, macrophages and B cells. This indicated that our prognostic panel could reflect alterations in the TIME. More experimental studies are warranted to illuminate the relationship between this prognostic panel and immune cells.

TAICs can damage tumor cells at an early stage, but tumors can evade surveillance by the immune system and suppress the cell-cytotoxic function of immunity by expressing immune checkpoints (Hanahan, 2022). The identified immune checkpoints included LAG3 (Guy et al., 2022), CD244 (Sun et al., 2021), CD274 (Cha et al., 2019), CD160 (Saleh et al., 2020), CTLA-4 (Rowshanravan et al., 2018), PDCD1 (Yi et al., 2022), PDCD1LG2 (The Cancer Genome Atlas Research Network, 2014) and TGFBR1 (Teicher, 2021). RECK was discovered to be significantly linked with CD244, CD160, PDCD1, PDCD1LG2, and TGFBR1 in our study. The expression of immune checkpoints could promote immune evasion in tumors. Targeting immune checkpoints is potentially effective in tumor treatment. For example, PD-1/PD-L1 antibodies (such as pembrolizumab and nivolumab) effectively function in GC therapies by blocking PD-1. This finding reveals that the expression of RECK is firmly associated with immune infiltration and immunological checkpoints.

In this study, we analysed all differential lncRNAs, miRNAs and mRNAs of gastric cancer using TCGA database. Then we innovatively constructed ceRNAs using lncRNAs, miRNAs and mRNAs and further constructed prognostic panel using LASSO algorithm. Finally, we obtained a novel prognostic model containing both lncRNA (PVT1), miRNA (hsa-mir-130a-3p) and mRNA (RECK). The RNAs included in our prognostic panel might function as novel potential GC therapeutic targets. More experimental researches are warranted to further investigate the mechanisms.

## 5 Conclusion

A novel three-element prognostic panel of gastric cancer based on the ceRNA network was established using the LASSO algorithm. This prognostic panel is firmly associated with clinical prognostic outcome, immune infiltration, clinicopathological features and immune checkpoints. This panel provides potential targets for GC therapy and more experimental research are needed.

## Data Availability

The datasets presented in this study can be found in online repositories. The names of the repository/repositories and accession number(s) can be found in the article/Supplementary Material.

## Abbreviations

ceRNA, competing endogenous RNA; DCs, dendritic cells; GC, gastric cancer; GO, Gene Ontology; HPA database, the Human Protein Atlas database; ICB, immune checkpoint blockade; OS, overall survival; KEGG, Kyoto Encyclopedia of Genes and Genomes; KM plot, Kaplan‒Meier Plotter; lncRNAs, Long noncoding RNAs; LASSO, the least absolute shrinkage and selection operator; miRNAs, MicroRNAs; MRE, miRNA response element; PFS, progression-free survival; PTEN, Phosphatase and tensin homolog; TAICs, tumor-antagonistic immune cells; TCGA, The Cancer Genome Atlas; TIME, the tumor immune microenvironment; TPICs, tumor-promoting immune cells; Tregs, regulatory T cells.
