# Supplementary material for: Comprehensive analysis to construct a novel immune-related prognostic panel in aging-related gastric cancer based on the lncRNA‒miRNA-mRNA ceRNA network
Source: Front Mol Biosci. 2023 May 15;10:1163977. doi: 10.3389/fmolb.2023.1163977 (PMC10226425; doi:10.3389/fmolb.2023.1163977)
Supplement: Supplementary file 1 [file Table1.DOCX]

Table 1. Correlation of PVT1 expression and clinical prognosis in gastric cancer in relation to various clinicopathological factors

| Clinicopathological characteristics | OS (n=875) | | | FPS (n=640) | | |
| --- | --- | --- | --- | --- | --- | --- |
|  | N | Hazard ratio | p value | N | Hazard ratio | p value |
| Sex |  |  |  |  |  |  |
| Female | 236 | 1.75(1.05-2.93) | 0.029^a^ | 201 | 2.48(1.69-3.63) | 1.5*10^-6^a^ |
| Male | 544 | 1.82(1.36-2.45) | 5.5*10^-5^a^ | 437 | 1.85(1.43-2.38) | 1.5*10^-6^a^ |
| T Stage |  |  |  |  |  |  |
| 1 | - | - | - | - | - | - |
| 2 | 241 | 1.88(1.09-3.23) | 0.021^a^ | 239 | 0.8(0.53-1.22) | 0.3 |
| 3 | 204 | 1.73(1.22-2.44) | 0.0017^a^ | 204 | 1.13(0.78-1.65) | 0.51 |
| 4 | 38 | 3.53(1.4-8.91) | 0.0047^a^ | 39 | 1.59(0.71-3.56) | 0.26 |
| N Stage |  |  |  |  |  |  |
| N0 | 74 | 2.23(0.96-5.17) | 0.055 | 72 | 1.9(0.64-5.6) | 0.24 |
| N+ | 422 | 1.56(1.2-2.03) | 0.00091^a^ | 423 | 1.18(0.9-1.53) | 0.23 |
| M Stage |  |  |  |  |  |  |
| M0 | 444 | 1.78(1.34-2.35) | 4.2*10^-5^a^ | 443 | 1.3(0.99-1.72) | 0.061 |
| M1 | 56 | 0.72(0.4-1.28) | 0.26 | 56 | 0.66(0.34-1.26) | 0.2 |
| Differentiation |  |  |  |  |  |  |
| Poorly | 165 | 2.16(1.3-3.59) | 0.0024^a^ | 121 | 0.76(0.49-1.2) | 0.25 |
| Moderately | 67 | 2.49(1.17-5.3) | 0.015^a^ | 67 | 0.68(0.34-1.34) | 0.26 |
| Well | 32 | 2.86(0.83-9.8) | 0.081 | - | - | - |
| Treatment |  |  |  |  |  |  |
| Surgery | 380 | 1.47(1.09-1.97) | 0.011^a^ | 375 | 1.38(1.03-1.84) | 0.028^a^ |
| 5-FU based adjuvant | 152 | 2.29(1.6-3.29) | 4.2*10^-6^a^ | 152 | 2.32(1.6-3.35) | 4.4*10^-6^a^ |
| others adjuvant | 76 | 1.55(0.62-3.9) | 0.34 | 80 | 1.76(0.78-3.97) | 0.17 |
| HER2 |  |  |  |  |  |  |
| Negative | 532 | 1.66(1.32-2.08) | 1.1*10^-5^a^ | 408 | 1.68(1.28-2.21) | 0.00019^a^ |
| Positive | 343 | 1.55(1.18-2.01) | 0.0012^a^ | 232 | 1.94(1.4-2.68) | 4.5*10^-5^a^ |

a means p < 0.05.
