# Supplementary material for: Comprehensive analysis to construct a novel immune-related prognostic panel in aging-related gastric cancer based on the lncRNA‒miRNA-mRNA ceRNA network
Source: Front Mol Biosci. 2023 May 15;10:1163977. doi: 10.3389/fmolb.2023.1163977 (PMC10226425; doi:10.3389/fmolb.2023.1163977)
Supplement: Supplementary file 2 [file Table2.DOCX]

Table 2.Correlation of hsa-miR-130a expression and clinical prognosis in gastric cancer in relation to various clinicopathological factors and immune cells.

| Clinicopathological characteristics | OS (n=7642) | | |
| --- | --- | --- | --- |
|  | N | Hazard ratio | p value |
| Sex |  |  |  |
| Female | 154 | 1.4(0.83-2.37) | 0.21 |
| Male | 277 | 1.84(1.13-2.98) | 0.013^a^ |
| Stage |  |  |  |
| 1 | 55 | 1.85(0.59-5.75) | 0.28 |
| 2 | 128 | 2.12(1.06-4.22) | 0.029^a^ |
| 3 | 178 | 1.45(0.94-2.23) | 0.09 |
| 4 | 43 | 1.84(0.69-4.95) | 0.22 |
| Grade |  |  |  |
| 1 | - | - | - |
| 2 | 153 | 2.16(1.26-3.71) | 0.004^a^ |
| 3 | 259 | 0.81(0.55-1.2) | 0.29 |
| 4 | - | - | - |
| Mutation burden |  |  |  |
| High | 215 | 2.04(1.12-3.72) | 0.017^a^ |
| Low | 210 | 1.45(0.96-2.19) | 0.075 |
| Neoantigen load |  |  |  |
| High | 73 | 2.7(0.95-7.67) | 0.052 |
| Low | - | - | - |
| *Restrict analysis based on cellular content* | | | |
| CD4+ memory T-cells |  |  |  |
| Enriched | 254 | 1.83(1.07-3.15) | 0.026^a^ |
| Decreased | 152 | 1.44(0.84-2.45) | 0.18 |
| CD8+ T-cells |  |  |  |
| Enriched | 204 | 1.51(0.9-2.55) | 0.21 |
| Decreased | 202 | 2.05(1.28-3.28) | 0.0023^a^ |
| Macrophages |  |  |  |
| Enriched | 217 | 1.46(0.86-2.49) | 0.16 |
| Decreased | 189 | 2.05(1.28-3.29) | 0.0023^a^ |
| B-cells |  |  |  |
| Enriched | 229 | 1.37(0.9-2.08) | 0.14 |
| Decreased | 177 | 2.3(1.23-4.31) | 0.0076^a^ |
| Eosinophils |  |  |  |
| Enriched | 304 | 1.74(1.21-2.5) | 0.0024^a^ |
| Decreased | 112 | 1.99(0.95-4.19) | 0.064 |
| Natural killer T-cells |  |  |  |
| Enriched | 260 | 1.61(1.08-2.41) | 0.018^a^ |
| Decreased | 146 | 1.37(0.83-2.28) | 0.22 |

a means p < 0.05.
