# Supplementary material for: Comprehensive analysis to construct a novel immune-related prognostic panel in aging-related gastric cancer based on the lncRNA‒miRNA-mRNA ceRNA network
Source: Front Mol Biosci. 2023 May 15;10:1163977. doi: 10.3389/fmolb.2023.1163977 (PMC10226425; doi:10.3389/fmolb.2023.1163977)
Supplement: Supplementary file 3 [file Table5.DOCX]

Table S1. Primer sequences of lncRNA, miRNA and mRNA for real-time polymerase chain reaction.

| Target | Sequence of primers |
| --- | --- |
| β-actin-Forward | CACCATTGGCAATGAGCGGTTC |
| β-actin-Reward | AGGTCTTTGCGGATGTCCACGT |
| RECK-Forward | CAACTCTGACAGCCCGACTTTGG |
| RECK-Reverse | AAGGAGGAGGGAGTGGCAAGAAG |
| hsa-miR-130a-3p Forward | CGCAGTGCAATGTTAAAAGGGCAT |
| lncRNA PVT1-Forward | CCAGTGGATTTCCTTGCGG |
| lncRNA PVT1-Reverse | CATCTTGAGGGGCATCTTTTTA |
