# Supplementary material for: Comprehensive analysis to construct a novel immune-related prognostic panel in aging-related gastric cancer based on the lncRNA‒miRNA-mRNA ceRNA network
Source: Front Mol Biosci. 2023 May 15;10:1163977. doi: 10.3389/fmolb.2023.1163977 (PMC10226425; doi:10.3389/fmolb.2023.1163977)
Supplement: Supplementary file 4 [file Table3.DOCX]

Table 3. Correlation of RECK expression and clinical prognosis in gastric cancer with various clinicopathological factors

| Clinicopathological characteristics | OS (n=875) | | | FPS (n=640) | | |
| --- | --- | --- | --- | --- | --- | --- |
|  | N | Hazard ratio | p value | N | Hazard ratio | p value |
| Sex |  |  |  |  |  |  |
| Female | 236 | 1.66(1.14-2.42) | 0.0079^a^ | 201 | 1.5(0.99-2.27) | 0.054 |
| Male | 544 | 1.22(0.96-1.56) | 0.098 | 437 | 1.19(0.92-1.54) | 0.18 |
| T Stage |  |  |  |  |  |  |
| 1 | - | - | - | - | - | - |
| 2 | 241 | 1.8(1.18-2.75) | 0.0058^a^ | 239 | 2.03(1.17-3.54) | 0.01^a^ |
| 3 | 204 | 1.26(0.91-1.83) | 0.15 | 204 | 1.26(0.91-1.77) | 0.17 |
| 4 | 38 | 3.36(1.36-8.28) | 0.0053^a^ | 39 | 2.65(1.17-5.99) | 0.015^a^ |
| N Stage |  |  |  |  |  |  |
| N0 | 74 | 2.29(0.92-5.68) | 0.066 | 72 | 2.19(0.88-5.41) | 0.083 |
| N+ | 422 | 2.06(1.57-2.69) | 7.9*10^-8^a^ | 423 | 1.86(1.44-2.41) | 1.7*10^-6^a^ |
| M Stage |  |  |  |  |  |  |
| M0 | 444 | 1.8(1.36-2.38) | 3.2*10^-5^a^ | 443 | 1.6(1.22-2.1) | 0.00055^a^ |
| M1 | 56 | 2.13(1.17-3.88) | 0.011^a^ | 56 | 1.68(0.92-3.08) | 0.088 |
| Differentiation |  |  |  |  |  |  |
| Poorly | 165 | 0.79(00.5-1.25) | 0.31 | 121 | 1.19(00.75-1.87) | 0.46 |
| Moderately | 67 | 1.43(0.74-2.77) | 0.28 | 67 | 1.42(0.74-2.75) | 0.29 |
| Well | 32 | 2.39(0.87-6.54) | 0.081 | - | - | - |
| Treatment |  |  |  |  |  |  |
| Surgery | 393 | 158(1.18-2.13) | 0.0022^a^ | 375 | 1.52(1.1-2.11) | 0.0099^a^ |
| 5-FU based adjuvant | 152 | 0.62(0.42-0.91) | 0.013^a^ | 152 | 0.58(0.39-0.84) | 0.0041^a^ |
| others adjuvant | 76 | 3.38(1.35-8.5) | 0.0059^a^ | 80 | 2.96(1.33-6.62) | 0.0055^a^ |
| HER2 |  |  |  |  |  |  |
| Negative | 532 | 1.42(1.12-1.8) | 0.0037^a^ | 408 | 1.42(1.08-1.85) | 0.01^a^ |
| Positive | 343 | 1.52(1.15-2) | 0.0028^a^ | 232 | 1.2(0.85-1.69) | 0.3 |

a means p < 0.05.
