# Supplementary material for: Comprehensive analysis to construct a novel immune-related prognostic panel in aging-related gastric cancer based on the lncRNA‒miRNA-mRNA ceRNA network
Source: Front Mol Biosci. 2023 May 15;10:1163977. doi: 10.3389/fmolb.2023.1163977 (PMC10226425; doi:10.3389/fmolb.2023.1163977)
Supplement: Supplementary file 5 [file Table4.DOCX]

Table 4. Correlation analysis between RECK and gene markers of immune cells in TIMER.

| Description | Gene markers | Cor | p |
| --- | --- | --- | --- |
| TAM | CCL2 | 0.546 | *** |
|  | CD68 | 0.252 | *** |
|  | IL10 | 5.54 | *** |
| M1 | IRF5 | 0.291 | *** |
|  | PTGS2 | 0.268 | *** |
| M2 | CD163 | 0.523 | *** |
|  | VSIG4 | 0.496 | *** |
| B cell | CD19 | 0.402 | *** |
|  | CD79A | 0.406 | *** |
| T cell | CD3D | 0.307 | *** |
|  | CD3E | 0.345 | *** |
|  | CD2 | 0.381 | *** |
| CD8+ T cell | CD8A | 0.345 | *** |
|  | CD8B | 0.261 | *** |
| Monocyte | CD86 | 0.468 | *** |
|  | CSF1R | 0.613 | *** |
| Neutrophils | MS4A4A | 0.574 | *** |
|  | CEACAM8 | 0.086 | 0.08 |
|  | ITGAM | 0.544 | *** |

Notes: * represents p less than 0.05, ** represents p less than 0.01, *** represents p less than 0.001.
